# Could the Combination of eGFR and mGPS Facilitate the Differential Diagnosis of Age-Related Renal Decline from Diseases? A Large Study on the Population of Western Sicily

**DOI:** 10.3390/jcm12237352

**Published:** 2023-11-28

**Authors:** Miriam Carella, Annamaria Porreca, Cinzia Piazza, Francesco Gervasi, Daniele Magro, Marika Venezia, Raffaella Lo Verso, Giuseppe Vitale, Annalisa Giusy Agnello, Letizia Scola, Tommaso Silvano Aronica, Carmela Rita Balistreri

**Affiliations:** 1 Complex Operative Unit of Clinical Pathology, ARNAS Civico Di Cristina e Benfratelli Hospitals, 90127 Palermo, Italy; miriam.carella@edu.unito.it (M.C.); tommasosilvano.aronica@arnascivico.it (T.S.A.); 2 Department of Medical, Oral Science and Biotechnology, University “G. d’Annunzio” Chieti-Pescara, 66100 Chieti, Italy; porreca.annamaria@gmail.com; 3 Specialized Laboratory of Oncology, ARNAS Civico Di Cristina e Benfratelli Hospitals, 90127 Palermo, Italy; francesco.gervasi@arnascivico.it; 4 Cellular and Molecular Laboratory, Department of Biomedicine, Neuroscience and Advanced Diagnostics (Bi.N.D.), University of Palermo, 90134 Palermo, Italy; daniele.magro@unipa.it (D.M.); marika.venezia@unipa.it (M.V.); letiza.scola@unipa.it (L.S.)

**Keywords:** renal age-related decline, kidney disease (KD), eGFR, mGPS

## Abstract

The assessment of renal function is critical to diagnosing and managing renal age-related decline, disease (KD), and failure, which are prevalent in the elderly population. The glomerular filtration rate (GFR) is widely used as an indicator of kidney function, but its direct measurement is challenging, as are its age and gender caveats. This makes difficult the differential diagnosis between age-related physiological decline and KD and/or failure. Currently, the inflammation-based modified Glasgow prognostic score (mGPS) is emerging as a promising biomarker of several inflammatory acute/chronic diseases. In this study, the large variability of eGFR with age and gender was evaluated as the association of eGFR values with mGPS levels. A population of 57,449 adult participants (age ≥ 18 years) was enrolled. Appropriate circulating biomarkers were measured to detect eGFR and mGPS values. The data obtained demonstrated a significant decrease in eGFR in men vs. women across the four selected age classes (18–40, 40–60, 60–80, 80–100 years); eGFR classes were significantly associated with mGPS (*p* < 0.001), as were age classes and gender with mGPS categories. Accordingly, the percentage of people having an mGPS score = 2 significantly increased across the eGFR classes: with an 11% in the G1/eGFR class needed to achieve 44% in G5/eGFR. Thus, the combination of mGPS with eGFR could represent the best benchmark risk model for the differential diagnosis of kidney disease from the age-related eGFR reduction.

## 1. Introduction

The phenomenon of aging represents one of the most significant social transformations of the twenty-first century, being a major issue of public health, because it is closely linked to an increased prevalence and incidence of a large array of chronic inflammatory diseases and conditions, such as acute/chronic kidney diseases (CKD) and failure [1,2]. Accordingly, it is imperative to identify biomarkers that can facilitate their prevention and management [3]. KD organizations and societies recommend the use of the estimated glomerular filtration rate (eGFR) [4,5] for their diagnosis. The eGFR index is considered the best overall index of kidney function in health and disease. Its eGFR values cannot be quantified directly and require the assessment of the clearance of either exogenous or endogenous filtration markers. Precisely, eGFR is obtained through a mathematic equation, which needs to detect the creatinine (CRE) or cystatin C levels and incorporate demographic factors, i.e., age, sex, and ethnicity [5,6]. Values of estimated GFR (eGFR) of <60 mL/min/1.73 m^2^ identify CKD in adults [6]. However, this fixed threshold shows a low appropriateness in the CKD diagnosis, since it does not consider the physiological decrease in eGFR with the advancement of age, as mostly debated in the last years [6,7,8,9,10,11,12,13,14,15]. It may, indeed, lead to underdiagnosis in young individuals and overdiagnosis in elderly individuals. Accordingly, eGFR physiologically decreases with kidney aging [6,7,8,9,10,11,12,13,14,15]. It reports an eGFR annual decline having a variability ranging from 0.3 to 2.6 mL/min per 1.73 m^2^. This is related to increased nephrosclerosis associated with the nephron loss during aging [16]. Accordingly, healthy adults aged 18–29 years to 70–75 years show a nephron loss of about 48%, and this knowledge is crucial because it is associated with increased mortality [16].

Furthermore, eGFR values appear to be influenced not only by age but also by sex/gender. Higher baseline eGFR values associated with a faster eGFR decline with age were detected more in men than in women (0.92 vs. 0.75) in a large population of 12,062 participants, with 58.7% being women, from the Rotterdam study conducted by Chaker and coworkers in 2021 [1]. This also implies amending the fixed threshold for gender, the well-recognized sexual dimorphism of kidney aging [17]. 

Based on current evidence, it is possible to affirm that age and gender appear among the major determinants of kidney function decline, and this implies a revised definition of KD accompanied with revised values of eGFR. Some advances have been achieved with a new CKD definition adapted with age and based on new eGFR thresholds of 75, 60, and 45 mL/min/1.73 m^2^ across the age classes ranging from 40, 40–64, to 65 years or older, respectively [12,17]. However, it is not yet clear which of the definitions allows us to identify true cases of CKD from those without, and, therefore, whether the revised cutoffs really allow us to distinguish age-related functional decline from true CKD or from failure and whether sex/gender should be included in a future, more standardized definition relating to the new thresholds [12,18]. This is also leading to the consideration of new biomarkers. A recent study, based on a modest number of CKD patients, has interestingly proposed to use the inflammation-based modified Glasgow prognostic score (mGPS) [19] in CKD management, given by the combination of C reactive protein (CRP) values with albumin levels. mGPS represents, however, the most used risk score in oncology, proving ulterior evidence on the fundamental role of inflammation and nutritional decline in cancer, even if it is well recognized in other diseases, such as CKD [20].

Based on the observations described above, we wanted to test and confirm the variability of eGFR values according to age classes and sex in a very large Western Sicilian population, represented by 57,449 adult participants (age ≥ 18 years). Moreover, we evaluated the association between eGFR classes and mGPS and the relationship between age classes and gender with the mGPS categories. 

## 2. Methods 

### 2.1. Study Design, Sources, and Population

We enrolled a sample of individuals selected by using the linked laboratory and administrative dataset from the A.R.N.A.S. Civico Di Cristina Benfratelli Hospitals. Precisely, 57,449 adult participants (age ≥ 18 years; see Table 1) were encompassed and admitted for medical examinations between the second half of 2021 and 31 December 2022. No upper limit of age for inclusion criteria was used. However, we included in the analysis individuals whose serum creatinine levels were measured and who were followed up at least once during the observation period. eGFR was calculated using the CKD-EPI 2021 equation without a race coefficient [6]. The institutional ethics review boards at the Universities of Palermo and the A.R.N.A.S. Civico Di Cristina Benfratelli Hospitals approved this study with a waiver of participant consent because of the retrospective study design and the secondary use of routinely collected administrative data.

### 2.2. Detection of Circulating Biomarkers 

Blood samples were obtained from all the individuals enrolled in the study for detecting CRE levels, as well as some inflammatory variables, including White Blood Cells (WBC), Neutrophils, Basophils, Eosinophils, Lymphocytes, Monocytes, Monocyte Distribution Width (MDW), Red Cell Distribution Width (RDW), Procalcitonin (PCT), and CRP. Moreover, we also assessed and considered two other parameters related to inflammation: the Neutrophils-to-Lymphocytes ratio (NLR) and the mGPS [19,21]. Furthermore, Albumin (ALB), Creatine Phosphokinase (CPK), Alkaline Phosphatase (ALP), and the Albumin-to-Creatinine ratio for kidney injury evaluation were also evaluated. For their detection, the routine methods and failures of the Complex Operative Unit of Clinical Pathology, ARNAS Civico Di Cristina e Benfratelli Hospitals, were used. They are described in detail in the eMethods of the Supplement. 

### 2.3. Statistical Analysis

Continuous variables were reported as means ± standard deviations (SD), and categorical variables were presented as absolute frequencies (%). Missing values are also reported. Pearson’s chi-square test was used to investigate the association among categorical variables. Pearson r correlation coefficients were estimated to evaluate the relationship between eGFR and the other quantitative variables. A multinomial multivariate logistic regression model was used to predict eGFR Classes and mGPS classes using Age in class and Gender as fixed factors. The odds ratios (ORs), 95% Confidence interval (CI), and *p*-value are reported. A *p*-value < 0.05 was considered statistically significant. Statistical analysis was performed using the R environment for statistical computing and graphics version 3.5.3 (R Foundation for Statistical Computing, Vienna, Austria).

## 3. Results

### 3.1. Baseline Characteristics 

**Figure 1:**
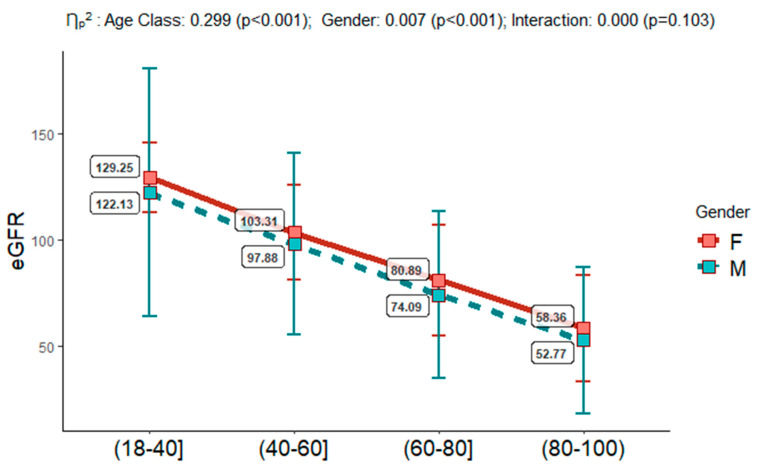
eGFR (estimated Glomerular Filtration Rate) mean ± SD (Standard Deviation) by Age Classes and Gender. Ƞ_p_^2^ = partial eta squared, and *p*-value derived from two-way factorial analysis of variance (ANOVA).

We collected 57,449 samples (Female = 29,329 (51.1%); Male = 28,120 (48.9%)), who were stratified by the following age classes: (18–40) n = 14,789, (40–60) n = 16,595, (60–80) n = 19,747, and (80–100) n = 6318 (see Table 1). eGFR values were assessed as described above and eGFR classes were detected. Interestingly, we observed that only 2.94% of the study population belonged to the G5/eGFR class, and 18.9% were of the mG2/mGPS category. The eGFR estimation, into the four age groups (18–40, 40–60, 60–80, 80–100), and in females vs. males, confirmed its documented reduction across the age classes, with higher mean values in females with respect to males (see Figure 1). According to our overall data, we detected an increase in inflammatory biomarkers, such as CRP, PCT, and the NLR, with higher values in the oldest-people class (see Table 1). The NLR was validated in numerous studies as a very sensitive indicator of infection, inflammation, and sepsis and may be considered an independent risk factor for KD progression in CKD patients [22,23].

### 3.2. Association of eGFR with the Inflammatory and Damage Circulating Biomarkers 

**Figure 2:**
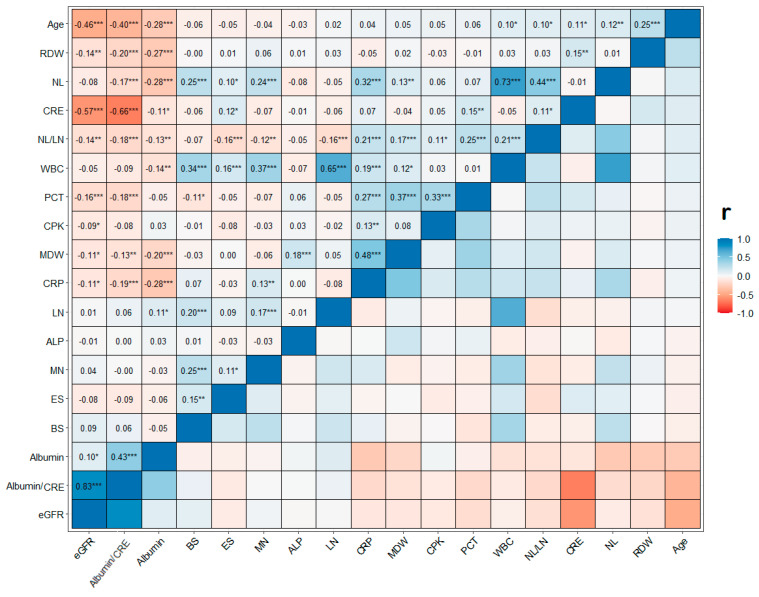
The correlation matrix (CM) of Pearson’s (r) correlation coefficient and listwise deletion methods. Significance level codes: *** *p* < 0.001, ** *p* < 0.01, and * *p* < 0.05. eGFR = estimated glomerular filtration rate, CRE = Creatinine, WBC = White Blood Cell, RDW = Red Blood Cells Distribution Width, NL = Neutrophils, LN = Lymphocytes, MN = Monocytes, ES = Eosinophils, BS = Basophils, MDW = Monocytes Distribution Width, CRP = C Reactive Protein, PCT = Procalcitonin, CPK = Creatine Phosphokinase, ALP = Alkaline Phosphatase.

Using eGFR in its continuous nature, we performed a correlation analysis to assess the relationship with the inflammatory and damage circulating biomarkers. As shown in Figure 2, The correlation analysis revealed a statistically significant, albeit weak, correlation between eGFR and inflammatory biomarkers. eGFR presented a weak, statistically significant, and positive correlation with Albumin (r = 0.10 and *p* < 0.05) and a weak, statistically significant, and negative correlation with CRP (r = −0.11, *p* < 0.05), MDW (r = −0.11, *p* < 0.05), PTC (r = −0.16, *p* < 0.001), CPK (r = −0.09, *p* < 0.05), RDW (r = −0.14, *p* < 0.01), and NLR (r = −0.14, *p* <0.01). The serum CRE and albumin-to-serum-CRE ratio were strongly correlated with the eGFR parameter (r = −0.57 and r = 0.83, *p* < 0.001, respectively).

### 3.3. eGFR-Based CKD Risk Definition

To better stratify risk in the population studied, we used the eGFR in the categorical form. The six eGFR categories are: G1 = 90 mL/min/1.73 m^2^ or higher: the normal range; G2 = 60–89 mL/min/1.73 m^2^: may mean early kidney disease; G3a = 45 to 59 mL/min/1.73 m^2^: mild to moderate loss of kidney function; G3b = 30 to 44 mL/min/1.73 m^2^, G4 = 15 to 29 mL/min/1.73 m^2^, and G5 = less than 15 mL/min/1.73 m^2^: kidney failure. The association of age classes and gender with different combinations of kidney dysfunction traits was investigated by multivariate multinomial logistic regression analysis (see Table 2). The subjects aged between 18 and 40 years had statistically significantly lower OR values for developing G2–G5 pathological conditions with respect to the oldest people (age class = 80–100 years), given that the other variables in the model are held constant. For age classes, there was evidence of a consistently lower risk of one for all eGFR classes with respect to G1. To be female also resulted in reducing the risk to be G2–G5 with respect to G1 (*p* < 0.001), given that the other variables in the model are held constant. 

### 3.4. mGPS Categories and Risk for CKD in Different Age Classes 

**Figure 3:**
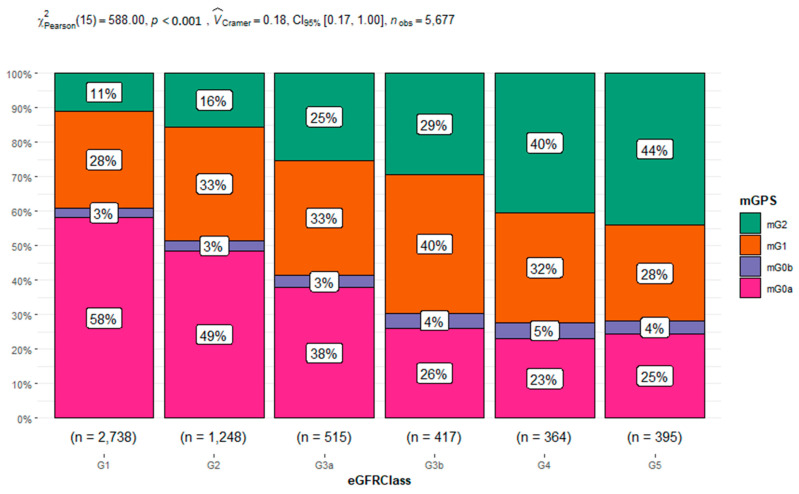
Distribution of inflammation-based modified Glasgow prognostic scores (mGPS) by chronic kidney disease stage eGFR Classes. The Chi squared test evaluated the association between variables.

We assessed the association between the mGPS and eGFR. The Chi-squared test showed a statistically significant association between the variables with a *p* < 0.001 (see Figure 3). Moreover, we found that 61% of the eGFR class = G1 presented an mGPS score of 0, and only 11% belonged to the eGFR class = G1 with an mGPS score of 2. A total of 29% of people belonging to eGFR class = G5 presented an mGPS score of 0, and 44% presented an mGPS score of 2. In addition, we studied the relationship between age classes and gender and mGPS (see Table 3) by using a multinomial multivariate logistic regression model for mGPS and age classes and gender as fixed factors. The results obtained demonstrated that the subjects aged between 18 and 40 years had a higher risk for mGPS0a, mGPS0b, and 1 with respect to the oldest people (age class = 80–100 years), with a *p* < 0.001. To be female also appeared to increase the risk of having an mGPS0 score rather than an mGPS2 score with an OR of 1.22 and a *p* < 0.009.

## 4. Discussion

The phenomenon of aging represents one of the most significant social transformations of the twenty-first century, representing the major cause of dangerous health challenges in the current era [21]. As with any other age-related chronic disease, CKD shows an increasing prevalence with an advancing age, as well as an increase in kidney failure cases accompanied by an augmented demand for dialysis and/or kidney transplantation in the old population [21,24,25]. This implies the imperative request to identify more appropriate criteria and biomarkers for an accurate kidney function evaluation, which possibly varies in relation to various age classes. Accordingly, the use of eGFR fixed thresholds is under discussion, which appear to show diverse caveats, including the overestimation of the CKD burden in the old population, overdiagnosis, and unnecessary interventions in many old people having only an age-related loss of eGFR. Consequently, eGFR as a first-line tool for estimating kidney function in real-world practice requires some revaluations, which first consider both the effect of age-related changes on the eGFR and its sex/gender-related variability. CKD presents sex disparities in the pathophysiology, as well as in the epidemiology, clinical manifestations, and disease progression [25,26,27].

The current eGFR is detected by using CRE values and equations derived from younger populations (precisely, under 65 years of age), and the application of other equations can lead to different eGFR results, affecting the CKD categories and severity classification [6,7]. This has led some researchers to suggest that these limitations can in part be reduced using equations based on cystatin C levels, even if non-GFR-related factors, such as obesity, inflammation, and a history of smoking, can affect circulating cystatin c values [28]. Considering this evidence, other research groups emphasize the estimation of the GFR using both CRE and cystatin C as biomarkers, since the use of this combined equation is more accurate than the use of either CRE or cystatin C alone in both older and younger patients [27,28,29,30]. 

Based on such evidence, in our study, including 57,449 adult participants (age ≥ 18 years), we first evaluated the variability of eGFR with age and gender by estimating its values in four age classes (18–40, 40–60, 60–80, 80–100) and for gender (females vs. males). In parallel, we also assessed the circulating levels of molecular/cellular inflammatory and damage biomarkers, currently documented to be related to CKD, including not only the albumin levels but also the recent NLR [18,22,23] and mGPS [19].

First, the data obtained confirmed the significant decrease in eGFR across the four age classes, with values starting from 129.25 to 122.13 in females vs. males in the 18–40 age class (corresponding to the G1 category with the fixed eGFR threshold), which slowly decrease in the 40–60 age class, maintaining higher levels in females than in males (103.31 vs. 97.68 mL/min per 1.73 m^2^). In the 60–80 age class, the values of eGFR were of 80.89–74.09 mL/min per 1.73 m^2^ (corresponding to the G2 category with the fixed eGFR threshold), arriving to values of 58.38–52.77 mL/min per 1.73 m^2^ in females vs. males, respectively, in the oldest people belonging to the 80–100 age class and corresponding to the G3a category of the fixed threshold. Our analysis evidenced that the age classes from 18 to 80 had OR values develop from G2 to G5 pathological conditions with respect to G1, which were significantly lower than those of the oldest age class of 80–100 years (*p* < 0.001).

Since our correlation analysis showed a weak association of eGFR with all the inflammatory biomarkers, eGFR appears to be not influenced by the inflammatory state. However, recent evidence demonstrates the key role of inflammation in CKD pathogenesis and progression [31]. Several mechanisms have been shown to contribute to an increased inflammatory response in patients with CKD, such as a decreased renal clearance of proinflammatory cytokines, oxidative and carbonyl stress, volume overload with endotoxemia, decreased levels of antioxidants, and an increased presence of comorbid conditions [31,32]. This is in accordance with current evidence on the promising role of other biomarkers related to inflammation in the progression of CKD, including those of tubule cell injury, e.g., KIM-1 and MCP-1, and those of mark tubule cell dysfunction (e.g., α1M and UMOD) [32], as well as mGPS [19]. The mGPS, encompassing both CRP and serum albumin, can capture not only the presence of the systemic inflammatory response but the nutritional status in pre-dialysis CKD patients [19]. Our data propose that the combination of eGFR and mGPS could be useful in the differential diagnosis of CKD from the eGFR reduction due to aging. We observed a statistically significant association between the mGPS and eGFR. We found that patients belonging to G1 presented a higher percentage of mGPS0, while patients belonging to G5 displayed a higher percentage of mGPS2. In addition, we analyzed the relationship between age classes and gender with the mGPS categories. We observed that patients belonging to the 18–40, 40–60, and 60–80 age classes had a higher risk for mGPS0a and 1 rather than mGPS2 with respect to the oldest people (age class = 80–100), with a *p* < 0.001. This statistical data was also presented by an OR > 1 but not statistically significant for the mGPS0b score. Only subjects aged between 18 and 40 years had a lower risk for mGPS2 with respect to the oldest people (age class = 80–100), with a *p* < 0.001. For the other age classes, this data was not statistically significant, possibly because mGPS0b and mGPS2 were both characterized by an albumin level < 3.5 g/dL. Moreover, we must consider that elderly individuals with a low skeletal muscle mass have been demonstrated to have an increased risk of albuminuria [28]. The reason for this relationship is not fully evident, even if age-related endothelial dysfunction characterized by an alteration in nitric oxide (NO) generation and increased oxidative stress is also suggested as a possible synergistic explanation, as well as hypertension [29].

Our data add more relevant evidence about the evolving concept of sex differences in CKD. We observed that the female sex reduces the risk of having the highest eGFR levels and increases the risk of having an mGPS0a score with respect to mGPS2. These sex differences could be explained by the direct effects of sex hormones on the kidney or sex differences in lifestyle factors [26].

## 5. Strengths and Limitations

This study has some strengths. First, we used population-based data from a geographically defined area served by a universal healthcare system. Thus, we analyzed a homogeneous population. Second, we examined a very large sample size. Third, we used validated algorithms to ascertain the presence or absence of comorbidities, and we applied recommended methods in the setting of competing risks. Fourth, we observed for the first time that the combination of mGPS with eGFR could represent the best parameter for differentiating age-related renal decline from KD and better identifying KD severity grades.

This study has several limitations. First, it relied on routinely collected data from people who accessed medical services. Second, we used the Inker et al. equation [6] to estimate kidney function. Because this equation has not been as well validated in older people as other equations, it could have led to inaccurate eGFR values for some people, although this inaccuracy should not have affected the findings regarding the relative risk of kidney failure in the study population. Third, we had insufficient information for assessing the clinical data of the population studied, even if they were in health. Although the findings require validation in other settings, we do not believe that these limitations pose a serious threat to the validity of our conclusions.

## 6. Conclusions

Based on the necessity to identify kidneys affected by CKD from aging kidneys [29] and the important role of inflammation in CKD pathogenesis and progression [30], we propose mGPS as a parameter to use in association with eGFR for improving the stratification of CKD patients. Other studies, likely multicenter, might help to better define the eGFR and mGPS relationship in CKD staging and propose a new CKD definition and guidelines.

## Figures and Tables

**Table 1:** Baseline Characteristics.

Characteristics	Overall	Age Classes							
		(18–40)		(40–60)		(60–80)		(80–100)	
		F	M	F	M	F	M	F	M
	n = 57,449	n = 9152	n = 5637	n = 8003	n = 8592	n = 8864	n=10,883	n = 3310	n = 3008
eGFRClass:									
G1	32,597 (57.7%)	8800 (97.4%)	4640 (83.5%)	6325 (80.1%)	4775 (56.4%)	4026 (46.4%)	3193 (29.8%)	477 (14.8%)	361 (12.2%)
G2	13,369 (23.7%)	190 (2.10%)	749 (13.5%)	1189 (15.1%)	2627 (31.1%)	2995 (34.5%)	3810 (35.6%)	1081 (33.5%)	728 (24.7%)
G3a	4255 (7.53%)	18 (0.20%)	72 (1.30%)	156 (1.98%)	496 (5.86%)	754 (8.69%)	1542 (14.4%)	668 (20.7%)	549 (18.6%)
G3b	2839 (5.02%)	11 (0.12%)	24 (0.43%)	81 (1.03%)	238 (2.81%)	429 (4.94%)	998 (9.32%)	532 (16.5%)	526 (17.8%)
G4	1785 (3.16%)	8 (0.09%)	21 (0.38%)	62 (0.79%)	126 (1.49%)	234 (2.70%)	573 (5.35%)	325 (10.1%)	436 (14.8%)
G5	1664 (2.94%)	12 (0.13%)	51 (0.92%)	84 (1.06%)	198 (2.34%)	240 (2.77%)	588 (5.49%)	142 (4.40%)	349 (11.8%)
mGPS:									
mGPS0a	2681 (47.2%)	435 (69.7%)	328 (63.0%)	371 (58.3%)	420 (52.7%)	421 (45.0%)	419 (36.6%)	167 (29.1%)	120 (27.1%)
mGPS0b	179 (3.15%)	9 (1.44%)	9 (1.73%)	11 (1.73%)	22 (2.76%)	37 (3.96%)	45 (3.93%)	29 (5.06%)	17 (3.84%)
mGPS1	1743 (30.7%)	161 (25.8%)	159 (30.5%)	180 (28.3%)	254 (31.9%)	269 (28.8%)	411 (35.9%)	162 (28.3%)	147 (33.2%)
mGPS2	1072 (18.9%)	19 (3.04%)	25 (4.80%)	74 (11.6%)	101 (12.7%)	208 (22.2%)	271 (23.6%)	215 (37.5%)	159 (35.9%)
CRE (mg/dL)	1.05 (1.03)	0.65 (0.34)	0.98 (0.79)	0.82 (0.74)	1.12 (1.15)	1.03 (0.98)	1.32 (1.28)	1.32 (1.06)	1.64 (1.46)
eGFR	94.3 (41.9)	129 (16.5)	122 (58.0)	103 (22.1)	97.9 (42.5)	80.9 (25.8)	74.1 (39.0)	58.4 (25.1)	52.8 (34.2)
WBC (×10^3^/µL)	8.99 (5.24)	9.41 (4.95)	9.20 (5.37)	8.26 (4.74)	9.07 (6.14)	8.63 (4.79)	8.93 (4.55)	9.83 (5.95)	9.46 (6.55)
RDW %	14.5 (2.11)	14.2 (1.87)	13.5 (1.43)	14.5 (2.24)	14.0 (1.77)	14.7 (2.27)	14.7 (2.12)	15.5 (2.47)	15.4 (2.35)
NL (×10^3^/µL)	6.21 (4.33)	6.62 (4.65)	6.07 (3.70)	5.45 (3.65)	6.09 (5.18)	5.94 (3.95)	6.23 (3.83)	7.37 (4.80)	6.90 (5.14)
LN (×10^3^/µL)	1.92 (2.16)	1.99 (0.80)	2.20 (3.48)	2.02 (1.27)	2.05 (1.39)	1.88 (2.53)	1.77 (1.94)	1.60 (3.22)	1.62 (3.28)
NL/LN	4.72(6.83)	4.15 (4.32)	3.86 (4.91)	3.54 (4.33)	4.02 (5.12)	4.67 (6.62)	5.32 (8.00)	7.93 (12.7)	7.59 (9.96)
MN (×10^3^/µL)	0.67 (0.96)	0.64 (0.28)	0.72 (0.57)	0.60 (1.75)	0.71 (0.87)	0.62 (0.53)	0.72 (1.06)	0.71 (0.75)	0.76 (1.00)
ES (×10^3^/µL)	0.14 (0.19)	0.11 (0.16)	0.16 (0.18)	0.13 (0.16)	0.16 (0.23)	0.13 (0.17)	0.15 (0.23)	0.10 (0.14)	0.13 (0.18)
BS (×10^3^/µL)	0.05 (0.08)	0.04 (0.07)	0.05 (0.06)	0.05 (0.13)	0.05 (0.05)	0.05 (0.06)	0.05 (0.05)	0.05 (0.08)	0.04 (0.06)
MDW (SDV)	19.2 (3.20)	19.3 (2.66)	18.4 (2.81)	18.9 (2.79)	18.6 (3.05)	19.5 (3.35)	19.1 (3.30)	20.3 (3.88)	20.3 (4.21)
CRP (mg/dL)	2.57 (5.80)	1.36 (3.73)	1.38 (3.93)	1.78 (4.92)	1.96 (5.14)	2.85 (6.13)	3.46 (6.79)	4.10 (6.93)	4.34 (7.13)
PCT (µg/L)	5.55 (19.3)	1.19 (2.75)	1.83 (8.69)	6.51 (22.4)	3.67 (14.8)	5.30 (19.1)	6.73 (22.6)	5.93 (16.2)	7.77 (23.7)
Albumin (g/dL)	3.95 (0.60)	3.95 (0.47)	4.45 (0.57)	4.10 (0.55)	4.10 (0.64)	3.88 (0.62)	3.84 (0.64)	3.54 (0.61)	3.53 (0.60)
CPK (U/L)	202 (942)	118 (254)	346 (1631)	165 (1141)	229 (1102)	150 (492)	205 (765)	183 (684)	194 (591)
ALP (U/L)	26.4 (71.1)	18.8 (45.4)	32.5 (90.0)	23.7 (57.2)	33.8 (95.0)	24.6 (54.0)	27.7 (72.7)	26.1 (74.2)	28.5 (85.1)
Albumin/CRE	1.03 (2.29)	2.80 (3.50)	0.73 (1.88)	1.01 (2.34)	0.61 (1.62)	0.73 (1.86)	0.55 (1.45)	0.68 (1.55)	0.51 (1.25)

Descriptive statistics are expressed as means (Standard Deviation) for continuous variables and as absolute frequencies (%) for categorical variables. The total of available cases is reported by variables (N). eGFR = estimated glomerular filtration rate, mGPS = modified Glasgow Prognostic Score, CRE = Creatinine, WBC = White Blood Cell, RDW = Red Blood Cells Distribution Width, NL = Neutrophils, LN = Lymphocytes, MN = Monocytes, ES = Eosinophils, BS = Basophils, MDW = Monocytes Distribution Width, CRP = C Reactive Protein, PCT = Procalcitonin, CPK = Creatine Phosphokinase, ALP = Alkaline Phosphatase.

**Table 2:** Multilevel multivariate logistic regression model for eGFR class using age classes and gender as fixed factors.

eGFR Class (Ref. Category G1)	Fixed Factors	OR	95% CI		*p*-Value
			Lower Limit	Upper Limit	
G2	Age Class:				
	(18–40) vs. (80–100)	0.03	0.03	0.03	<0.001
	(40–60) vs. (80–100)	0.14	0.12	0.15	<0.001
	(60–80) vs. (80–100)	0.39	0.35	0.42	<0.001
	Gender (F vs. M)	0.44	0.42	0.46	<0.001
	Age Class:				
G3a	(18–40) vs. (80–100)	0.00	0.00	0.01	<0.001
	(40–60) vs. (80–100)	0.03	0.03	0.04	<0.001
	(60–80) vs. (80–100)	0.19	0.17	0.21	<0.001
	Gender (F vs. M)	0.34	0.32	0.37	<0.001
G3b	Age Class:				
	(18–40) vs. (80–100)	0.00	0.00	0.00	<0.001
	(40–60) vs. (80–100)	0.02	0.02	0.02	<0.001
	(60–80) vs. (80–100)	0.13	0.12	0.15	<0.001
	Gender (F vs. M)	0.32	0.29	0.35	<0.001
G4	Age Class:				
	(18–40) vs. (80–100)	0.00	0.00	0.00	<0.001
	(40–60) vs. (80–100)	0.01	0.01	0.02	<0.001
	(60–80) vs. (80–100)	0.10	0.09	0.12	<0.001
	Gender (F vs. M)	0.28	0.25	0.31	<0.001
G5	Age Class:				
	(18–40) vs. (80–100)	0.01	0.01	0.01	<0.001
	(40–60) vs. (80–100)	0.03	0.03	0.04	<0.001
	(60–80) vs. (80–100)	0.16	0.14	0.18	<0.001
	Gender (F vs. M)	0.23	0.20	0.25	<0.001

OR = odds ratio, 95% CI = Confidence Interval.

**Table 3:** Multilevel multivariate logistic regression model for inflammatory-based modified Glasgow prognostic score (mGPS) categories using age classes and genders as fixed factors.

mGPS (Ref. mGPS2)	Fixed Factors	OR	95% CI		*p*-Value
			Lower Limit	Upper Limit	
mGPS0a	Age Class:				
	(18–40) vs. (80–100)	22.77	16.20	32.02	<0.001
	(40–60) vs. (80–100)	6.05	4.83	7.58	<0.001
	(60–80) vs. (80–100)	2.34	1.93	2.84	<0.001
	Gender (F vs. M)	1.22	1.05	1.42	0.009
	Age Class:				
mGPS0b	(18–40) vs. (80–100)	3.33	1.78	6.24	<0.001
	(40–60) vs. (80–100)	1.54	0.95	2.49	0.081
	(60–80) vs. (80–100)	1.40	0.95	2.06	0.092
	Gender (F vs. M)	1.02	0.74	1.41	0.889
mGPS1	Age Class:				
	(18–40) vs. (80–100)	8.76	6.18	12.43	<0.001
	(40–60) vs. (80–100)	2.96	2.34	3.73	<0.001
	(60–80) vs. (80–100)	1.69	1.40	2.05	<0.001
	Gender (F vs. M)	0.89	0.76	1.04	0.150

OR = odds ratio, 95% CI = Confidence Interval.

## Data Availability

Data sharing is not applicable; no new data were created or analyzed in this study.

## Supplementary Materials

The following supporting information can be downloaded at: https://www.mdpi.com/article/10.3390/jcm12237352/s1, eMethods [33,34,35,36,37].

## References

[1] van der Burgh A.C., Rizopoulos D., Ikram M.A., Hoorn E.J., Chaker L. (2021). Determinants of the Evolution of Kidney Function with Age. Kidney Int. Rep..

[2] Dybiec J., Szlagor M., Młynarska E., Rysz J., Franczyk B. (2022). Structural and Functional Changes in Aging Kidneys. Int. J. Mol. Sci..

[3] Balistreri C.R. (2018). Anti-Inflamm-Ageing and/or Anti-Age-Related Disease Emerging Treatments: A Historical Alchemy or Revolutionary Effective Procedures?. Mediat. Inflamm..

[4] Levey A.S., Coresh J., Tighiouart H., Greene T., Inker L.A. (2020). Measured and estimated glomerular filtration rate: Current status and future directions. Nat. Rev. Nephrol..

[5] Inker L.A., Collier W., Greene T., Miao S., Chaudhari J., Appel G.B., Badve S.V., Caravaca-Fontán F., Del Vecchio L., Floege J. (2023). A meta-analysis of GFR slope as a surrogate endpoint for kidney failure. Nat Med..

[6] Inker L.A., Eneanya N.D., Coresh J., Tighiouart H., Wang D., Sang Y., Crews D.C., Doria A., Estrella M.M., Froissart M. (2021). New Creatinine- and Cystatin C-Based Equations to Estimate GFR without Race. N. Engl. J. Med..

[7] Levey A.S., Grams M.E., Inker L.A. (2022). Use of eGFR in Older Adults with Kidney Disease. Reply. N. Engl. J. Med..

[8] Carnevale V., Tinti M.G. (2022). Use of eGFR in Older Adults with Kidney Disease. N. Engl. J. Med..

[9] Toyama T., Kitagawa K., Oshima M., Kitajima S., Hara A., Iwata Y., Sakai N., Shimizu M., Hashiba A., Furuichi K. (2020). Age differences in the relationships between risk factors and loss of kidney function: A general population cohort study. BMC Nephrol..

[10] Minutolo R., Gabbai F.B., Chiodini P., Provenzano M., Borrelli S., Garofalo C., Bellizzi V., Russo D., Conte G., De Nicola L. (2020). Sex Differences in the Progression of CKD Among Older Patients: Pooled Analysis of 4 Cohort Studies. Am. J. Kidney Dis..

[11] Ravani P., Quinn R., Fiocco M., Liu P., Al-Wahsh H., Lam N., Hemmelgarn B.R., Manns B.J., James M.T., Joanette Y. (2020). Association of Age With Risk of Kidney Failure in Adults With Stage IV Chronic Kidney Disease in Canada. JAMA Netw. Open.

[12] Liu P., Quinn R.R., Lam N.N., Elliott M.J., Xu Y., James M.T., Manns B., Ravani P. (2021). Accounting for Age in the Definition of Chronic Kidney Disease. JAMA Intern. Med..

[13] Noronha I.L., Santa-Catharina G.P., Andrade L., Coelho V.A., Jacob-Filho W., Elias R.M. (2022). Glomerular filtration in the aging population. Front. Med..

[14] Jaques D.A., Vollenweider P., Bochud M., Ponte B. (2022). Aging and hypertension in kidney function decline: A 10 year population-based study. Front. Cardiovasc. Med..

[15] Singh-Manoux A., Oumarou-Ibrahim A., Machado-Fragua M.D., Dumurgier J., Brunner E.J., Kivimaki M., Fayosse A., Sabia S. (2022). Association between kidney function and incidence of dementia: 10-year follow-up of the Whitehall II cohort study. Age Ageing.

[16] Baylis C. (2009). Sexual dimorphism in the aging kidney: Differences in the nitric oxide system. Nat. Rev. Nephrol..

[17] Delanaye P., Jager K.J., Bökenkamp A., Christensson A., Dubourg L., Eriksen B.O., Gaillard F., Gambaro G., van der Giet M., Glassock R.J. (2019). CKD: A call for an age-adapted definition. J. Am. Soc. Nephrol..

[18] Zahorec R. (2021). Neutrophil-to-lymphocyte ratio, past, present and future perspectives. Bratisl Lek Listy.

[19] Stefan G., Stancu S., Zugravu A., Capusa C. (2022). Inflammation-based modified Glasgow prognostic score and renal outcome in chronic kidney disease patients: Is there a relationship?. Intern. Med. J..

[20] Wu T.H., Tsai Y.T., Chen K.Y., Yap W.K., Luan C.W. (2023). Utility of High-Sensitivity Modified Glasgow Prognostic Score in Cancer Prognosis: A Systemic Review and Meta-Analysis. Int. J. Mol. Sci..

[21] Campisi J., Kapahi P., Lithgow G.J., Melov S., Newman J.C., Verdin E. (2019). From discoveries in ageing research to therapeutics for healthy ageing. Nature.

[22] Yoshitomi R., Nakayama M., Sakoh T., Fukui A., Katafuchi E., Seki M., Tsuda S., Nakano T., Tsuruya K., Kitazono T. (2019). High neutrophil/lymphocyte ratio is associated with poor renal outcomes in Japanese patients with chronic kidney disease. Ren. Fail..

[23] Mureșan A.V., Russu E., Arbănași E.M., Kaller R., Hosu I., Arbănași E.M., Voidăzan S.T. (2022). The Predictive Value of NLR, MLR, and PLR in the Outcome of End-Stage Kidney Disease Patients. Biomedicines.

[24] Bowe B., Xie Y., Li T., Mokdad A.H., Xian H., Yan Y., Maddukuri G., Al-Aly Z. (2018). Changes in the US Burden of Chronic Kidney Disease From 2002 to 2016: An Analysis of the Global Burden of Disease Study. JAMA Netw. Open.

[25] Ke C., Liang J., Liu M., Liu S., Wang C. (2022). Burden of chronic kidney disease and its risk-attributable burden in 137 low-and middle-income countries, 1990-2019: Results from the global burden of disease study 2019. BMC Nephrol..

[26] Conte C., Antonelli G., Melica M.E., Tarocchi M., Romagnani P., Peired A.J. (2023). Role of Sex Hormones in Prevalent Kidney Diseases. Int. J. Mol. Sci..

[27] Thurlow J.S., Joshi M., Yan G., Norris K.C., Agodoa L.Y., Yuan C.M., Nee R. (2021). Global Epidemiology of End-Stage Kidney Disease and Disparities in Kidney Replacement Therapy. Am. J. Nephrol..

[28] Yoo J.H., Kim G., Park S.W., Choi M.S., Ahn J., Jin S.M., Hur K.Y., Lee M.K., Kang M., Kim J.H. (2020). Effects of low skeletal muscle mass and sarcopenic obesity on albuminuria: A 7-year longitudinal study. Sci. Rep..

[29] Balistreri C.R. (2022). Promising Strategies for Preserving Adult Endothelium Health and Reversing Its Dysfunction: From Liquid Biopsy to New Omics Technologies and Noninvasive Circulating Biomarkers. Int. J. Mol. Sci..

[30] Trachtman H. (2020). Age-Dependent Definition of CKD. J. Am. Soc. Nephrol..

[31] Mihai S., Codrici E., Popescu I.D., Enciu A.M., Albulescu L., Necula L.G., Mambet C., Anton G., Tanase C. (2018). Inflammation-Related Mechanisms in Chronic Kidney Disease Prediction, Progression, and Outcome. J. Immunol. Res..

[32] Ix J.H., Shlipak M.G. (2021). The Promise of Tubule Biomarkers in Kidney Disease: A Review. Am. J. Kidney Dis..

[33] https://www.beckmancoulter.com/it/products/hematology/dxh-900.

[34] https://fardavar.com/.

[35] https://www.biomerieux-nordic.com/product/vidasr-3.

[36] https://diagnostics.roche.com/.

[37] Fan H., Shao Z.Y., Xiao Y.Y., Xie Z.H., Chen W., Xie H., Qin G.Y., Zhao N.Q. (2016). Comparison of the Glasgow Prognostic Score (GPS) and the modified Glasgow Prognostic Score (mGPS) in evaluating the prognosis of patients with operable and inoperable non-small cell lung cancer. J. Cancer Res. Clin. Oncol..

