# Supplementary material for: Could the Combination of eGFR and mGPS Facilitate the Differential Diagnosis of Age-Related Renal Decline from Diseases? A Large Study on the Population of Western Sicily
Source: J Clin Med. 2023 Nov 28;12(23):7352. doi: 10.3390/jcm12237352 (PMC10707716; doi:10.3390/jcm12237352)
Supplement: Supplementary file 1 [file jcm-12-07352-s001.zip › jcm-2730823-supplementary.pdf]

Supplemental Online Content

eMethods

eMethods

Detection of circulating biomarkers

White Blood Cells (WBC), Neutrophils, Basophils, Eosinophils, Lymphocytes, Monocytes, Monocyte Distribution Width (MDW) and Red Cell Distribution Width (RDW) were detected by the Unicel DxH 900 Series (Beckman Coulter) Complete Blood Count analysis through Coulter Principle and VCS 360 Technology according to the manufacturer's instructions [1].

Parameter	Method	Analytical Measuring Range	Operating Range	Units	CV (Repeatability)
WBC	Coulter Principle	0.050–400.000	0.000–999.999	10^3 cells/µL	(0.500 2.000) <5%<br>(5.000–10.000) <3%
NE#	VCS 360 Technology (Calculated)	0.000–400.000	0.000–600.000	10^3 cells/µL	<3.5%
LY#	VCS 360 Technology (Calculated)	0.000–400.000	0.000–600.000	10^3 cells/µL	<5%
MO#	VCS 360 Technology (Calculated)	0.000–400.000	0.000–600.000	10^3 cells/µL	<10%
EO#	VCS 360 Technology (Calculated)	0.000–400.000	0.000–600.000	10^3 cells/µL	SD 0.5 or 13.5% CV @WBC > 4.0x103 cells/µL
BA #	VCS 360 Technology (Calculated)	0.000–400.000	0.000–600.000	10^3 cells/µL	SD 0.5 @WBC > 4.0x103 cells/µL
RDW	Derived from RBC Histogram	10.00–40.00	0.00–70.00	%	<2.5%
MDW	VCS 360 technology	14.00-39.00	0.0 - 255.0	N/A	<10%

PCT was performed by VIDAS® 3 analyzer (Biomérieux) combining a one-step immunoassay sandwich method with a final fluorescent detection (ELFA, Enzyme-Linked Fluorescent Assay), according to the manufacturer's instructions., with 0.05 ng/mL as a limit of

quantification (LoQ) and a CV≤9.4% (repeatability). For PCT concentrations greater than 200 ng/mL sample can be diluted with Serum Free reagent by one 1:10 dilution, extending the measurement range up to 2000 ng/mL [2,3].

Clinical chemistry tests as Creatinine (CRE) for the eGFR index, Albumin (ALB), C Reactive Protein (CRP), Creatine Phosphokinase (CPK), Alkaline Phosphatase (ALP) were performed by cobas® 8000 modular analyzer series (Roche Diagnostics) according to the manufacturer's instructions [4].

**Clinical chemistry test**	**Method**	Analytical Measuring Range	Units	CV% (Repeatability)
Creatinine	kinetic colorimetric assay (Jaffé method)	0.17-24.9	mg/dL	<2.5
C Reactive Protein	particle-enhanced immunoturbidimetric assay	0.6-350	mg/L	<2.3
Creatine kinase	UV-test (equimolar quantities of NADPH and ATP are formed at the same rate)	7-2000	U/L	<0.7
Albumin	colorimetric assay	2-60	g/L	<1.2
Alkaline phosphatase	colorimetric assay	5-1200	U/L	<0.9

Modified Glasgow prognostic score

The detection of modified Glasgow Prognosis Score (mGPS) was based on the serum levels of albumin and C-reactive protein (CRP). It was calculated as follows [5]:

- mGPS0a score, patients with CRP ≤1.0 mg/ dL and albumin ≥3.5 g/dL;
- mGPS0b score, patients with CRP ≤1.0 mg/ dL and albumin <3.5 g/dL;
- mGPS1 score, patients with CRP >1.0 mg/ dL and albumin ≥3.5 g/dL;
- mGPS2 score, patients with CRP >1.0 mg/ dL and albumin <3.5 g/dL;

eMethods References

1. https://www.beckmancoulter.com/it/products/hematology/dxh-900
2. https://fardavar.com/
3. https://www.biomerieux-nordic.com/product/vidasr-3
4. https://diagnostics.roche.com/
5. Fan, H., Shao, ZY., Xiao, YY. *et al.* Comparison of the Glasgow Prognostic Score (GPS) and the modified Glasgow Prognostic Score (mGPS) in evaluating the prognosis of patients with operable and inoperable non-small cell lung cancer. *J Cancer Res Clin Oncol* **142**, 1285–1297 (2016). https://doi.org/10.1007/s00432-015-2113-0
